# Introduction to the programme guidance for the use of iodised salt in processed foods and its pilot implementation, strengthening strategies to improve iodine status

**DOI:** 10.1371/journal.pone.0274301

**Published:** 2023-10-12

**Authors:** Jacky Knowles, Karen Codling, Robin Houston, Jonathan Gorstein

**Affiliations:** 1 Iodine Global Network, Ontario, Canada; 2 Bill & Melinda Gates Foundation, Seattle, Washington, United States of America; Public Library of Science, UNITED KINGDOM

## Abstract

Efforts to achieve optimal iodine intake through salt iodisation have focussed primarily on iodisation of household salt. However, there is strong evidence that in most regions of the world, industrially processed foods and condiments are an increasingly important source of dietary salt. In this context The Iodine Global Network (IGN) and partners developed programme guidance to help national programme managers assess the potential contribution of widely consumed industrially processed foods and condiments to iodine intake. The programme guidance additionally aimed to facilitate better understanding of iodised salt use by the processed food industry, review existing salt iodisation legislation for inclusion of food industry salt, and investigate how regulatory monitoring of food industry practices could be strengthened if needed. To evaluate the utility of the guidance in practice and identify areas where it could be improved, the IGN requested expressions of interest to pilot test implementation. Five pilots were implemented in Kenya, North Macedonia, The Republic of Moldova, Sri Lanka and Thailand, with remote technical support from IGN. The pilots demonstrated how evidence from implementation could be used to strengthen existing salt iodisation initiatives. In particular, how modelling existing processed food intake data enhanced understanding of potential or actual iodised salt intake and provided an evidence base for strategic change, as well as encouraging alignment with salt reduction programmes. In summary, the guidance provided a useful framework for national teams to conduct a relatively rapid assessment of the existing programme for achieving optimal iodine nutrition and opportunities to strengthen it. National teams involved with the pilot implementation were highly engaged and motivated by the outcomes. The pilot implementation process resulted in the development of strategic recommendations nationally and provided invaluable feedback to IGN on the utility of the guidance, facilitating development of an improved version.

## Introduction

### Rationale for developing programme guidance on assessing the use of iodised salt in industrially processed foods

Universal salt iodisation (USI) is the fortification with iodine of all food-grade salt for human and animal consumption, including salt for food processing. It is recognised as a cost-efficient and effective strategy to prevent and control iodine deficiency [1,2]. Iodisation of all food-grade salt was proposed as an equitable intervention to provide dietary iodine in addition to existing, variable, sources of iodine, at a level that would move populations from possible insufficient intake among all or some groups, to optimal iodine status among all groups. There is strong evidence that this intervention can achieve this aim, if well-implemented [3,4].

As of November 2021, 126 countries have implemented mandatory legislation for iodisation of some form of food-grade salt (111 of these have legislation for household and processed food salt), while a further 21 countries have some form of voluntary legislation [5].

To date, efforts to achieve optimal iodine intake through salt iodisation have been primarily focussed on iodisation of household salt alone. This is reflected in existing global guidance and in the widespread use of the proxy indicator of > 90% households using salt iodised to national standards, to show achievement of USI [6]. There is now strong evidence that in most regions of the world dietary patterns are shifting towards increased consumption of salt-containing industrially processed foods and condiments [7–9]. However, even in countries where legislation for processed food salt exists, the presence and level of iodine in food industry salt is often not known and regulatory monitoring of food industry salt is typically not part of national efforts to achieve entire population access to iodised salt.

The Iodine Global Network (IGN) and partners therefore developed programme guidance to assess the use of iodised salt by the food industry and the potential contribution of widely consumed industrially processed foods to iodine intake from the use of iodised salt [10]. Implementation of the guidance will be particularly relevant in countries with wide-spread consumption of industrially processed foods.

The overall expected outcome from implementing the guidance will be national evidence-based recommendations to effectively initiate, strengthen, or sustain the inclusion of food industry salt in the salt iodisation strategy, as needed. Benefits of strengthening the salt iodisation strategy to include food industry salt include the following:

◾ Facilitate understanding of the likely impact on population iodine intake of adjusting salt iodine standards or the implementation of salt reduction policies.
◾ Protect present and future generations from iodine deficiency through supplying the appropriate amount of iodine, accounting for changes in dietary practices and salt industry iodisation practices that may affect whether main sources of quality-assured iodised salt are household salt and / or food industry salt.

This paper and the related programme guidance define industrially processed foods as both foods and condiments produced by food industries that purchase salt in bulk and produce foods with relatively wide market reach. Products are usually packaged and branded. IGN recognises that consumption of processed foods high in calories, fat, sugar and salt can be a risk factor for non-communicable diseases [11] and does not endorse or encourage consumption of industrially processed foods.

### Overview of the guidance development, content, and main tools

The guidance was developed by IGN and directs programme managers through a series of modules, using associated tools and other resources. The main purpose of each module of the programme guidance is summarised in Table 1. The guidance suggests starting with a listing of all known national data sources to identify widely consumed foods contributing to salt intake, for example, dietary intake surveys. These foods are typically either foods consumed in small amounts but with a high salt content, for example bouillon, or foods with lower salt content that are consumed at relatively high volume and / or frequently, for example bread and instant noodles. The listing examines sources of data to estimate typical national consumption and the salt content of identified foods produced in the country or imported, and investigate whether iodised salt is used in their manufacture. The next two modules (Module 2 and Module 3) provide background and context to the assessment. These Modules guide a review of data for current national and sub-national iodine status, and for household use of iodised salt, as well as existing legislation for salt iodisation and its enforcement, with particular regard to the food industry.

**Table 1:** Overview of the main purpose of each of the programme guidance modules.

Module number and title	Overview of purpose of the module
Module 1. Listing available data and information sources for use in the assessment.	Facilitates review and documentation of different available data sources that will be used in Module 4. These include household surveys, research studies, salt producer and trader records, processed food industry and government databases, and national food standards. The listing helps identify data sources required to identify widely consumed foods contributing to salt intake and to estimate their consumption and salt content. This process helps understand any major information gaps and establishes feasibility and expectations for the rest of the process.
Module 2. Situation analysis to determine the need to strengthen the salt iodisation strategy.	Facilitates an analysis of household use of iodised salt, iodine status among different population groups, and current knowledge about consumption of salt-containing industrially processed foods to assess whether sustaining or strengthening the use of iodised salt by the food industry, would increase the likelihood of achieving and/or sustaining optimal iodine status.
Module 3. Review of the current legislative and enforcement framework for the use of iodised salt in industrially processed foods.	Facilitates a high-level review of the legislative framework for salt iodisation and of supporting monitoring and enforcement responsibilities, to identify whether food industry salt is included in the existing legislative scope and where legislation and/or monitoring and enforcement systems may be strengthened.
Module 4. Assessment of the contribution of industrially processed foods to salt and iodine intake	Facilitates a process to identify widely consumed industrially processed foods, estimate the contribution of these foods to typical daily salt intake where consumption data for identified foods are available, and model the contribution to iodine intake if all salt is iodised. Where typical daily consumption data are unavailable, the assessment can be implemented based on serving size of identified processed foods. The process and output increase understanding of the likely impact of mandating the use of iodised salt in identified industrially processed foods products. The main assessment framework, which forms the basis for Module 4, is shown in Fig 1 below, for the scenario based on typical daily intake of household salt and identified processed foods.
Module 5. Review the enabling environment for iodisation of all food-grade salt	Facilitates a review of national enabling factors required to achieve the goal of optimal population iodine status, for example, legislation, salt iodine levels and food control protocols. An understanding of the strengths and weaknesses of the enabling environment for salt iodisation may be used to develop recommendations for strategic change.
Module 6. Write a report for policy makers with recommendations to strengthen the salt iodisation strategy, as needed	Facilitates development of a national report presenting the assessment outcomes to policy makers and food industry leaders. Including recommendations that can be used to advocate for a strengthened, refocused, programme to achieve optimal iodine status through iodisation of all food-grade salt, as is nationally relevant.

**Fig 1:**
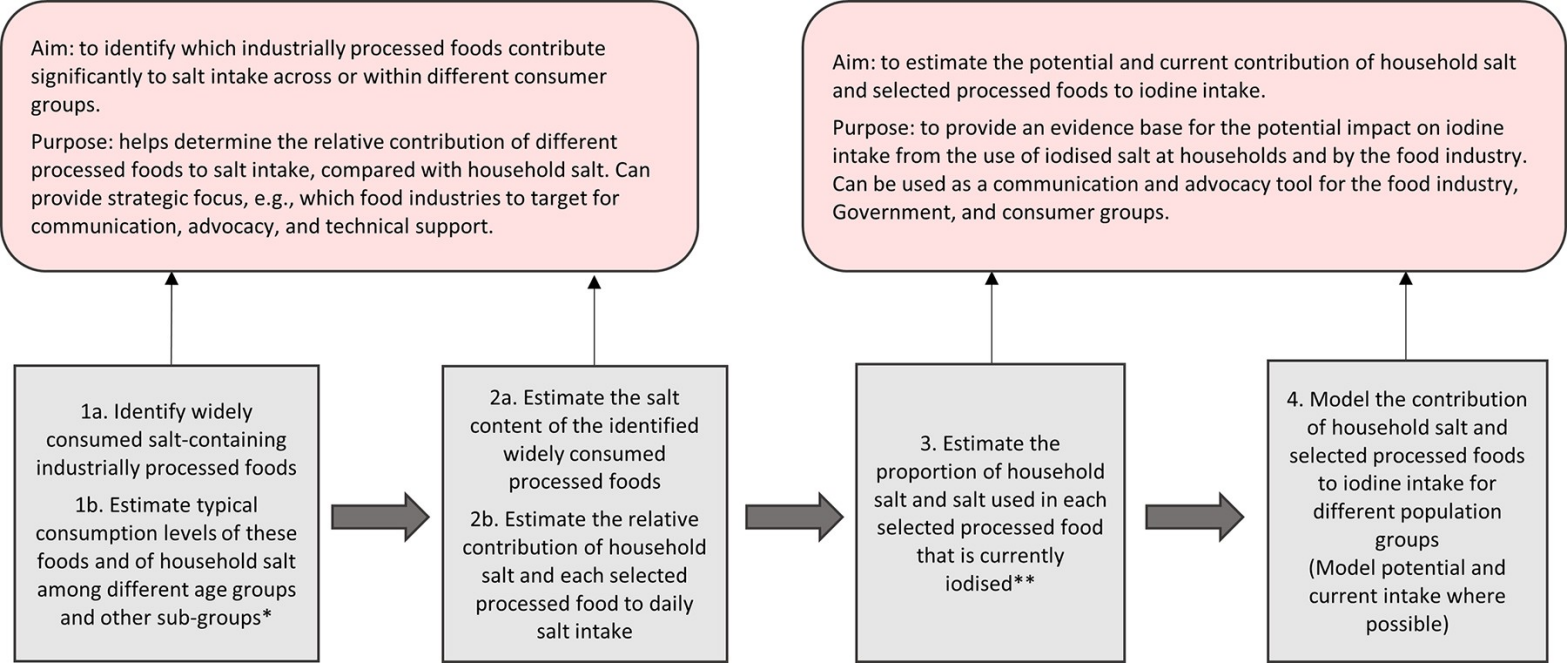
Key guidance framework from Module 4 –Assessing the contribution of industrially processed foods to salt and iodine intake (based on typical per capita intake data). * Example population groups often reported in surveys: School-age children, women of reproductive age, lactating women, pregnant women. Example sub-groups: Geographic region, residence type, socio-economic. ** Based on an assumption that, if iodised, salt will be iodised according to national standards. Where no information is available on the use of iodised salt, it is still possible to model potential iodine intake if all food grade salt is iodised, Step 4.

The main part of the assessment is in Module 4 which guides the user through a framework, described in Fig 1, for an assessment based on typical per capita intake of household salt and selected processed foods.

The assessment aims to estimate the potential contribution to iodine intake from typical daily consumption of household salt and of identified processed foods, based on data for estimated consumption and the average percent salt content of each food. The potential contribution to iodine intake from salt in these foods assumes that all salt is iodised to national standards and accounts for 30% loss of iodine in the final product. Where data are available to estimate the current proportion of household and food industry salt that is iodised, then the same assessment can also be conducted for estimated current (instead of potential) iodine intake from these products. Where consumption data are unavailable, the same assessments can be implemented based on average serving size of identified processed foods.

**Fig 2:**
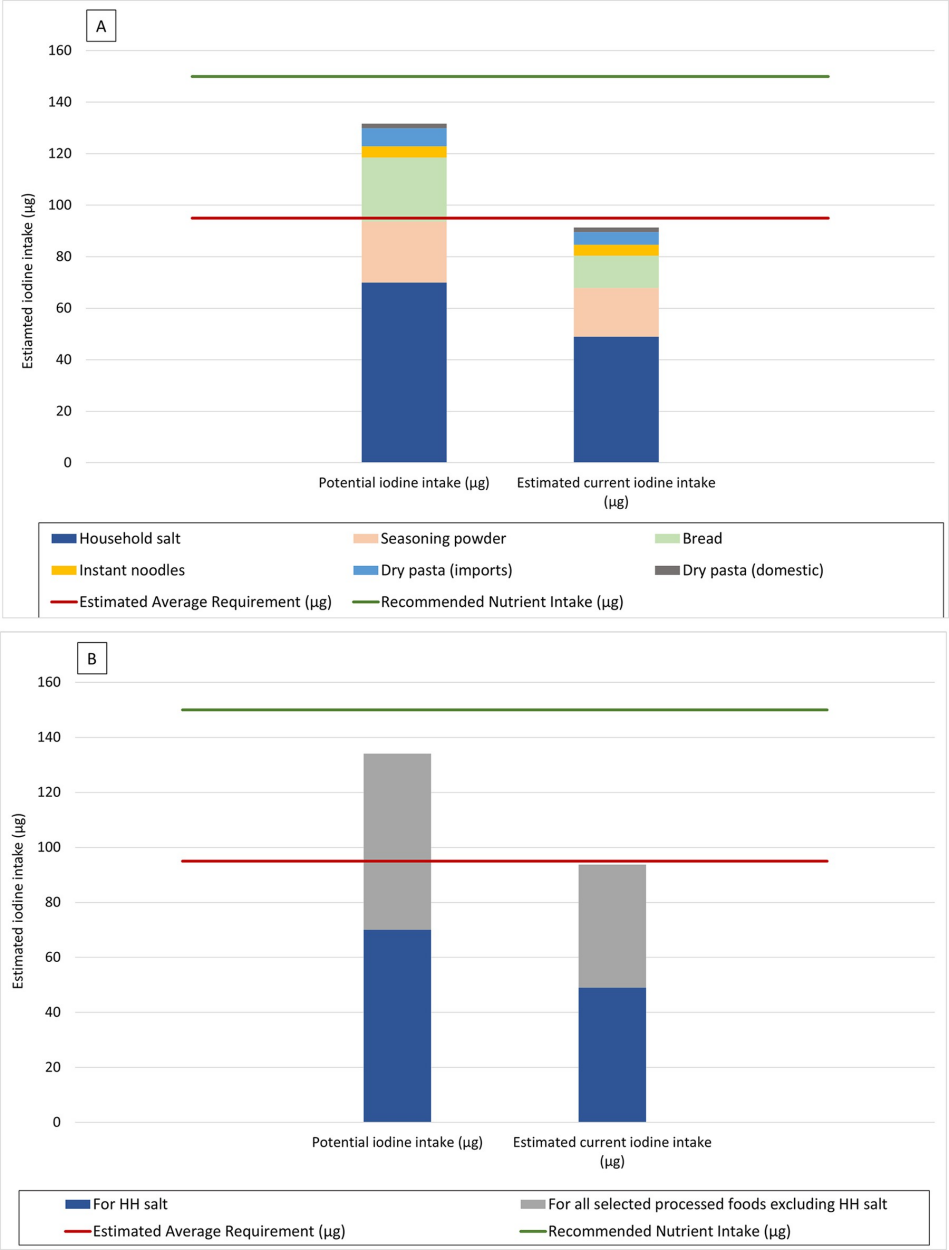
Potential and estimated current iodine intake from household salt and five selected processed foods, relative to the EAR and RNI for iodine for non-pregnant adults. **Based on example data.** (A). Iodine intake from household salt and from iodised salt in each of the selected processed food. (B). Iodine intake from household salt and from iodised food industry salt for the five selected processed foods combined. Illustrated using EAR = 95μg and RNI = 150 μg iodine for non-pregnant adults. Potential iodine intake represents iodine intake from salt in each product if all salt iodised according to national standards, and accounting for a 30% loss of iodine in the final product. Estimated current iodine intake represents iodine intake from the estimated percent of household salt and salt in each product currently iodised, with iodisation according to national standards, and accounting for a 30% loss of iodine in the final product.

**Fig 3:**
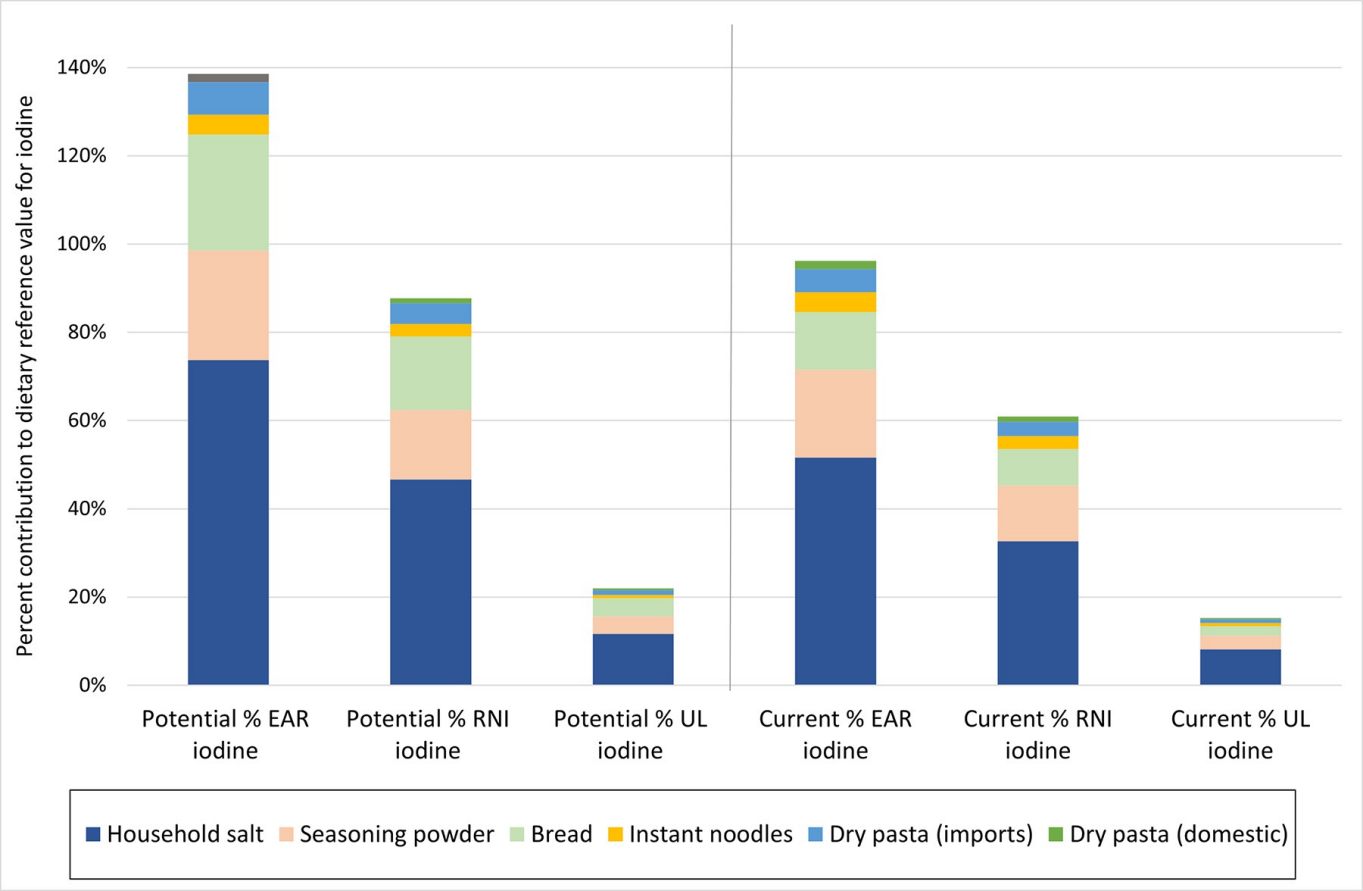
Potential and estimated current iodine intake from household salt and five selected processed foods, as a percentage of the EAR, RNI, and UL for iodine for non-pregnant adults. Based on example data. Illustrated using EAR = 95μg, RNI = 150 μg and UL = 600 μg iodine for non-pregnant adults. Potential iodine intake represents iodine intake from salt in each product if all salt iodised according to national standards, and accounting for a 30% loss of iodine in the final product. Estimated current iodine intake represents iodine intake from the estimated percent of household salt and salt in each product currently iodised, with iodisation according to national standards, and accounting for a 30% loss of iodine in the final product.

The estimated contribution to household salt and food industry salt to iodine intake can be expressed in relation to the estimated average requirement (EAR) for iodine, percent recommended nutrient intake (RNI) for iodine and percent of the tolerable upper limit for iodine (UL). Tools accompanying the programme guidance provide options to show the data as μg iodine in relation to these dietary reference values, or as a percent contribution to the EAR, RNI and UL for iodine; for each food individually, or as aggregated values for all identified processed foods compared with the contribution from household salt. Dietary reference values for iodine in the guidance are taken from a 2019 paper that proposed a set of harmonised values for multiple nutrients including iodine. [12] Figs 2A, 2B and 3 are examples of some ways that the data from the assessment could be shown. These Figs 2 and 3 are based on dummy typical per capita intake among non-pregnant adults of 4g household salt, 3g seasoning powder or bouillon, 95g bread, 7.7g instant noodles, and 25g pasta (20g imported, 5 g domestically produced).

Tools and resources were developed alongside the guidance to facilitate its implementation and documentation. The main tools (a Word table and Excel files)have been updated based on the outcome of the pilot and are available on the Iodine Global Network site [10]. The Word table prompts the implementation team to document key information for each stage, which provides a helpful record from which to write up a final report of the complete process and to replicate the assessment later if the situation changes. The Excel files have worksheets with pre-existing formulae to produce the required outputs described for Module 4. Outputs are reliant on national inputs for salt iodine standards, selected salt-containing processed foods, estimated typical intake or serving size amount for a specific population group, percent salt content of each food, and the percent salt used in manufacture of estimated to be iodised for each food. Different Excel tools were developed for the following 3 situations: (Option 1) where estimates of per capita consumption of selected processed foods are available, (Option 2) where processed foods to include in the assessment could be identified however no consumption data were available, but their serving sizes were known, and (Option 3) where data were available only for estimated quantities of food grade salt distributed for retail (as household salt) and for use by the food industry. The assessment using Option 1 produces the most helpful evidence to illustrate the impact of using iodised salt in selected processed foods and, therefore, for deciding on the need for strategic change.

The first draft of the guidance was based on experience from similar assessments in a number of countries [13] and was completed in 2018. In order to evaluate the utility of the guidance in practice, and to identify areas where it could be strengthened before finalisation, it was proposed to pilot test its implementation by national teams in countries from different regions. An additional aim of the pilot was to help determine the level of technical support required for effective future implementation in other countries. This paper serves as an introduction to both this special collection and to the programme guidance, including a high-level review of the pilot process and outcomes. Additional papers in this collection are authored by four of the five national teams who implemented the pilots and these provide more detailed information about national experiences.

The process of recruiting pilot national teams, reviewing the outcomes, and adapting lessons learned to produce an improved final version of the Guidance, benefitted from appraisal and inputs from an Advisory Partner Group. This group included representation from: Institute of Nutrition of Central America and Panama (INCAP), The Global Alliance for Improved Nutrition (GAIN), Nutrition International (NI), TechnoServe, The George Institute for Global Health, UNICEF, Unilever, and USAID.

## Methods

### Pilot implementation of the guidance

IGN sent out a request in late 2018 for national teams to submit an Expression of Interest (EOI) to pilot the guidance and to identify opportunities to strengthen the processed food component of national salt iodisation strategies, as needed. The EOI was communicated to national programme managers via IGN regional coordinators and in collaboration with global partners such as UNICEF, GAIN, INCAP and NI. The two intended outputs from the pilot were: 1. Findings and conclusions from implementation that would be helpful to understand and drive programme changes at the national level, and 2. Evaluation of the guidance including usability; clarity of instructions, examples and tools; appropriateness of content; and assessment of resources needed for implementation.

Selection of pilot locations aimed to identify national programmes most likely to benefit from the process. Pre-determined selection criteria included having:

◾ Mandatory legislation for salt iodisation.
◾ An existing national coordinating system, body, or organisation for salt iodisation/food fortification in general.
◾ Relatively recent data for the following: Household iodised salt use, iodine status among at least one population group, consumption or other data to identify which salt-containing, industrially processed, foods were widely consumed and contributing to salt intake.
◾ A willingness to apply opportunities identified during the pilot to strengthen the national salt iodisation strategy.
◾ A proposed focal point for the pilot exercise who had relevant experience and skills.

In addition to these criteria, IGN felt it would be helpful to support Expressions of Interest from countries with different situations for salt iodisation, different dietary practices, and from a range of regions. The five countries selected to pilot the guidance (along with the IGN designated region) were: Kenya (Eastern and Southern Africa), North Macedonia (Western and Central Europe), Republic of Moldova (Eastern Europe and Central Asia), Sri Lanka (South Asia), Thailand (South East Asia and the Pacific).

National teams were requested to establish a working group to coordinate implementation of the steps and methodologies detailed in the programme guidance. IGN provided national teams with regular online technical assistance (team calls and emails) and a low-level of funding to support some of the implementation costs. The period of pilot implementation of the guidance was March to December 2019.

## Results and discussion

This special collection includes papers written by the relevant national teams that report details on the foods selected, the assessment process, and on national findings for Moldova [14], North Macedonia [15], Sri Lanka [16], and Thailand [17]. The national team in Kenya did not submit a paper for inclusion, therefore the findings are not discussed in this collection overview paper.

### Summary of findings and recommendations from national pilots

Results for the two intended aims of the pilot were as follows.

**Fig 4:**
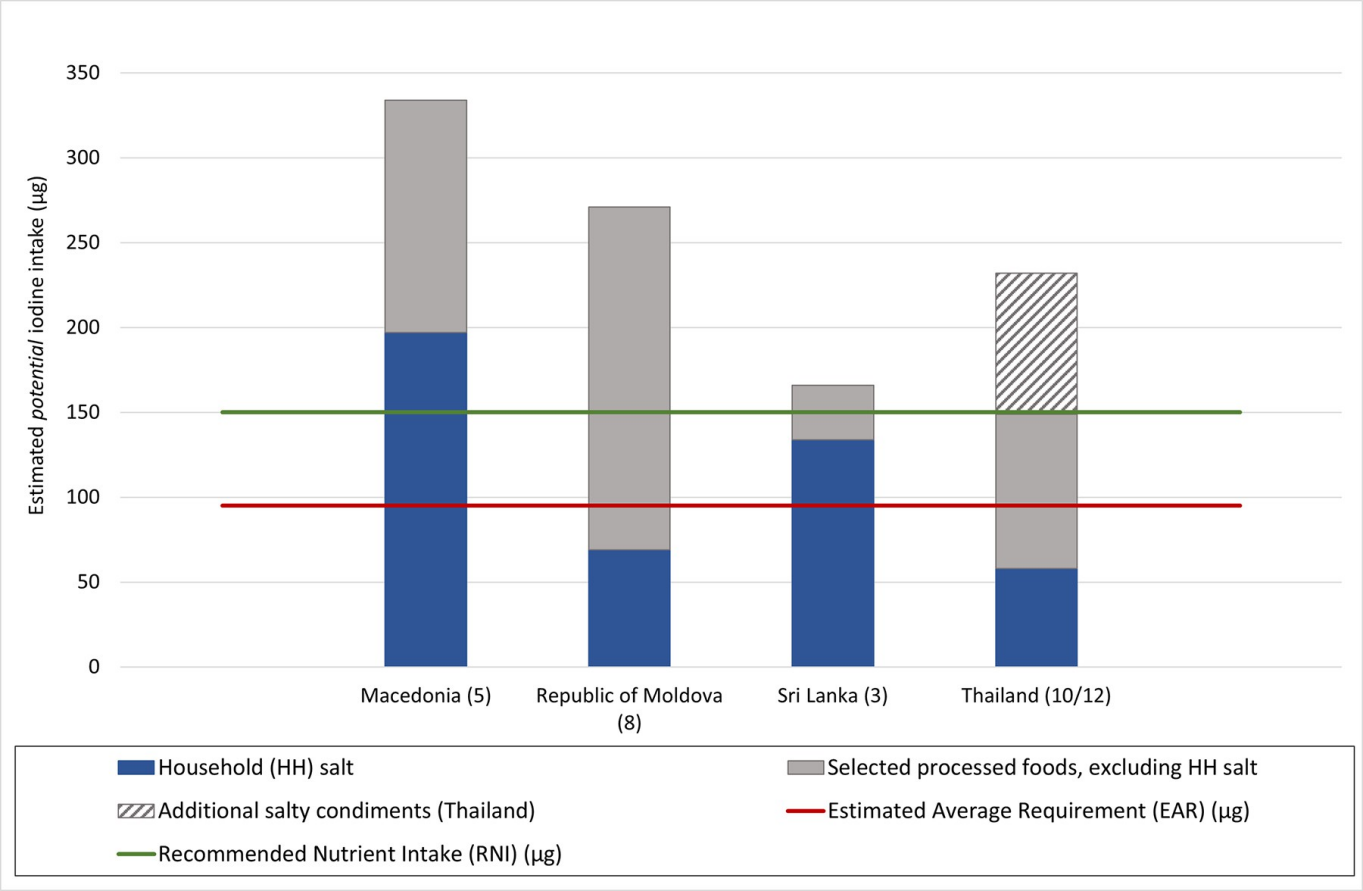
Potential iodine intake from iodised household salt and iodised salt in selected industrially processed foods, based on typical adult daily consumption and 100% salt iodised to national standards. Potential daily iodine intake assumes all food grade salt iodised to mean of national standard, with a 30% loss of iodine in the final product. Country name (n) = number of processed foods included in the national assessment, not including household salt. The Thai data shows results for top 10 processed foods contributing to daily salt intake (solid bar), and for an additional two salty condiments (hashed bar).

1. Implementation of the guidance resulted in national programme findings and recommendations for each of the pilot countries, a summary of these is included in Table 2. National outcomes varied considerably according to context. Fig 4. gives a summary of the potential contribution to the EAR and RNI for iodine for adults from iodised household salt and iodised salt in the selected processed foods for each of North Macedonia, the Republic of Moldova, Sri Lanka and Thailand, based on the potential for 100% salt iodisation.
2. Changes and additions to the current programme guidance were made based on feedback from national teams and on lessons learned by the technical support team during the process. Revised versions of the guidance and supporting tools are available from The Iodine Global Network. [10] Recommendations for additional modules to consider in future versions was a further outcome of the process.

**Table 2:** Summary of main findings and recommendations from each of the pilot countries.

Country	Programme Status^a^	Key Findings	Key Recommendations
North Macedonia [15]	Mandatory legislation for iodisation of all food grade salt. Household use of iodised salt was above 99%. [18] Iodine status among school age children and pregnant women was adequate. [18]	The salt iodisation strategy has been successful, including salt for the domestic food industry, and achieving optimal population iodine status. There was a data gap for iodised salt use in imported processed foods, which have a relatively large market share. There was high population total salt intake. Consumption of iodised household salt and iodised salt from five selected processed foods was estimated to contribute to approximately 350% of the EAR, 220% of the RNI and 55% of the UL for iodine for non-pregnant adults.	Sustain regular assessment of iodine status to verify that different groups have optimal iodine status. Maintain and strengthen inspection, enforcement of, and engagement with, the food industry. Review and expand the National Iodine Committee membership to include customs and the food industry. Work with non-communicable diseases to implement salt reduction alongside salt iodisation.
Republic of Moldova [14]	Mandatory legislation for iodisation of all food grade salt. Household use of iodised salt was 57% of households with salt with > 15mg/kg iodine, and 77% of households with salt with some iodine. [19] Iodine status among adult men and women was adequate. [19]	Legislative and regulatory documents regarding the (mandatory) use of iodised salt by the food industry have been misinterpreted in some instances and thus not enforced. There was a data gap for iodised salt use in imported processed foods, which have a relatively large market share There was high population total salt intake. Consumption of iodised household salt and iodised salt from eight selected processed foods had the potential to contribute to approximately 240% of the EAR, 150% of the RNI and 40% of the UL for iodine for non-pregnant adults. Estimates based on understanding of the current use of iodised salt were that household salt and salt in the eight processed foods met approximately 120%, 75% and 20% of the EAR, RNI and UL for iodine for non-pregnant adults respectively. With 50–55μg iodine intake per day from each of iodised household salt and iodised salt in bread.	Resolve ambiguities in the current legislation for salt iodisation and clearly communicate the requirements for the use of iodised salt by the food industry. Strengthen inspection, enforcement and engagement with the food industry. Improve assessment of the consumption of salt-containing processed foods and of iodine status among different groups to enable stronger monitoring of impact. To improve strategic approaches for salt iodisation through strengthening inter-sectoral links, for example with the public health agency, the national food and nutrition steering committee, the food industry, customs, and the non-communicable disease prevention committee.
Sri Lanka [16]	Mandatory legislation for iodisation of all food grade salt. Median household salt iodine content was between 18mg/kg to 27.5mg/kg by province. [20] Iodine status was adequate among school age children and pregnant women. [20]	Industrially processed foods had a limited market; bread, biscuits & dried fish were included in the assessment. Dried fish production was exempt from using iodised salt, it is not categorised as a processed food and uses the cheapest salt available. Estimated that 90% RNI iodine for adults and 55% RNI for pregnant women can be met from iodised table salt and iodised salt in bread and biscuits. Estimated current iodine intake from typical consumption of iodised household salt and iodised salt in bread and biscuits contributes to approximately 115% of the EAR, 75% of the RNI and 20% of the UL for iodine for non-pregnant adults. These figures would reduce to 80%, 75% and 20% respectively if 30% salt reduction was achieved across all products.	The salt iodisation strategy, including current salt iodine levels, is appropriate to maintain optimal iodine status. Re-focus regulatory monitoring efforts on salt and food industries, instead of household salt at retail level. Monitor the potential impact of the national salt reduction policy on iodine intake through regular assessment of iodine status among different groups.
Thailand [17]	Mandatory legislation for iodisation of all food grade salt. Alternative legislation existed for fish sauce and soy-based sauces and salty brine allowing for direct iodisation of the finished product. [21,22] Household use of iodised salt was 73% (with estimated 65% to 70% using salt with iodine >15mg/kg). [23] Iodine status was adequate among children 3 to 5 years of age and among elderly adults over 60 years of age. Status was borderline sufficient among pregnant women [24].	Multiple sources of relevant data were available. Estimated current iodine intake from typical consumption of iodised household salt and the iodised salt in the top 12 most widely consumed processed foods (including fish sauce and soy-based sauces and salty brine) contributed to approximately 240% of the EAR, 150% of the RNI and 40% of the UL for iodine for non-pregnant adults. Fish sauce and soy-based sauces and salty brine had the potential to contribute to approximately 100%, 50% and 15% of the adult EAR, RNI and UL for iodine respectively, if iodised salt was used in their production (generally non-iodised salt is used). Iodine intake from household salt, iodised salt in selected processed foods (not including fish sauce and soy-based sauces and salty brine), and from non-salt iodine sources such as rice, milk and eggs, had the potential to meet over 100% of the RNI for iodine for non-pregnant adults and approximately 80% of the RNI for pregnant women.	Strengthen enforcement of iodised salt use by the food industry and of direct iodisation of salty condiments, for which different regulations apply. Monitor the impact of improved regulatory monitoring through regular assessment of iodine status among adults, children and especially among pregnant women. Maintain the policy of direct addition of iodine to fish sauce and soy-based sauces and salty brine until the regular assessment of iodine status among pregnant women and other groups confirms sustained optimal iodine status.

^a^ Legislation status information is from the Global Fortification Data Exchange [25].

### Cross cutting issues from national pilots

Cross cutting findings from pilot implementation of the guidance can be summarised in terms of opportunities and challenges. Implementing the guidance created sufficient evidence in all pilot countries to identify opportunities for future strategic change. The assessment demonstrated that iodised salt in processed foods has the potential to contribute significantly to iodine intake in North Macedonia, the Republic of Moldova and Thailand, and, to a lesser extent, in Sri Lanka. Where processed foods are not made with iodised salt (either because it is not legislated or not enforced), iodine intake was considerably lower than it could be potentially.

The implementations process highlighted gaps in data, some of which are described in Table 2, that resulted in recommendations for future data collection. The modelled national data enhanced understanding of potential or actual iodised salt sources and provided an evidence base for strategic change and alignment with salt reduction programmes. For example, the total estimated salt intake from selected products along with household salt was used to understand the potential contribution to population iodine intake from iodising food industry salt. In addition, the modelling could be used to examine the potential impact of successful salt reduction policies on salt and potential iodine intake from different products and to estimate the impact of changing salt iodisation standards on potential iodine intake. All teams concluded that, although salt reduction or changing salt iodine levels were possible future strategic aims, an important first step would be to strengthen regular assessment of the adequacy of population iodine intake to assess the need for and monitor the impact of any future strategic changes. National teams all reported that assessment outcomes provided opportunities to engage and collaborate more closely with relevant Government departments and the food industry to mandate or strengthen enforcement for the use of iodised salt by the food industry; and with experts working on non-communicable disease prevention, specifically on salt reduction.

The EOI submitted by national teams were based on the understanding that data on food production, import, and / or consumption were available to conduct the assessment in Module 4. However, one of the main challenges identified during the process was a lack of available data in this or other areas. Suggested sources of relevant data, described in the first module of the guidance, were expected to provide the following type of information for industrially processed foods: typical household and individual consumption of different identified salt-containing foods; which food products use the largest volume of salt in their manufacture, for domestic consumption; whether products are made with iodised or non-iodised salt; and an estimate of market share and regional distribution of food products identified for inclusion in the assessment. The type and applicability of data for each purpose varied between the four pilot countries included in this paper and is described in more detail in the nationally-authored papers in this collection. The avaibility of relevant data influenced the level of detail and applicability of modelling outcomes, since in some cases more generalised data, such as food composition information from neighbouring countries, had to be used.

The exception to this problem of lack of consumption data was the Thai study, where the team had access to consumption data for a wide range of salt-containing industrially processed food products and were, therefore, able to generate a fairly comprehensive model for potential iodine intake from these foods if all food industry salt was iodised.

In Sri Lanka, not many widely consumed salt-containing foods manufactured on an industrial scale were identified. Bread, dried fish, and biscuits were the main products. Consumption of street foods is common in Sri Lanka but since these foods are expected to be primarily made with retail/household salt it was not considered relevant to include them. The current version of the guidance does not separate out the contribution of street foods to salt and potential iodine intake. However, further analysis of this issue has been listed for potential addition to a subsequent version.

North Macedonia and the Republic of Moldova teams faced the challenge of trying to obtain data on the use of iodised salt in imported processed foods products, which have a large market share in these countries. Models were therefore based on best available information about salt iodisation practices in the country of origin, which were documented as part of the process. Deeper investigation of how to obtain reliable information about the volume of imported processed foods according to county of origin, and of related food industry practices, is also listed as a potential addition to a subsequent version of the guidance.

All national teams concluded that stronger inspection and enforcement of salt iodisation was needed, with particular focus on ensuring the use of iodised salt by the processed food industry. In some countries, protocols for inspection of salt producers and importers existed and were implemented, however, inspection and enforcement of processed food manufacturers were often not included in national protocols.

## Conclusions

The revised version of the guidance, incorporating the lessons and recommendations from the pilot together with the outcome of a review by the Advisory Partner Group, was released in 2021. A summary report from the pilot implementation process and national outcomes are included as case studies, along with a description of data and methods used in previous case studies that followed a similar process.

The pilots demonstrated that implementation of the guidance required ‘judgement calls’ or assumptions to be made when there were gaps in the relevant data. The pilot teams reported that it was useful to discuss such decisions and other parts of the process with the IGN technical support team. Based on this, IGN will be applying for funding to provide some level of technical support for national teams from other countries to implement the guidance in the future, as well as to develop a training package and train regional resource personnel to provide more local support.

In summary, the guidance provided a useful framework for national teams to conduct a relatively quick assessment of the existing programme for achieving optimal iodine nutrition and of opportunities to strengthen it. National teams involved with the pilot implementation were highly engaged and motivated by the outcomes, as well as providing invaluable feedback to IGN on the utility of the version of the guidance used. These factors made the pilot implementation process successful in terms of developing national strategic recommendations and in the development of an improved version of the guidance and its associated tools.
